# Identification of novel CRESS-DNA viruses in the human vaginal microbiome

**DOI:** 10.3389/fmicb.2026.1790643

**Published:** 2026-04-17

**Authors:** Ziyuan Dai, Qiang Lu, Mingzhong Sun, Hongmei Chen, Yuchen Jiang, Taotao Yu, Zhipeng Wang, Yungang Wang, Rong Zhu, Yuqing Han

**Affiliations:** Department of Clinical Laboratory, Yancheng Third People's Hospital, The Affiliated Hospital of Jiangsu Medical College, Affiliated Hospital 6 of Nantong University, Yancheng, Jiangsu, China

**Keywords:** Cressdnaviricota, human, metagenomics, phylogenetic analysis, vaginal virome

## Abstract

**Introduction:**

Circular replication-associated protein (Rep)-encoding single-stranded DNA (CRESS-DNA) viruses are widely distributed across diverse hosts and environments, yet their diversity within the human vaginal virome remains poorly characterized. This study aimed to investigate the presence, diversity, and evolutionary relationships of CRESS-DNA viruses in the human vaginal niche.

**Methods:**

Viral metagenomic sequencing was performed on 24 pooled vaginal swab libraries derived from women with and without vaginitis. After host sequence removal and quality control, de novo assembly and viral identification were conducted. Candidate viral genomes were curated based on genomic features, followed by functional annotation, phylogenetic analysis using Rep protein sequences, and genome-wide pairwise nucleotide identity comparisons.

**Results:**

A total of five CRESS-DNA viral genomes were identified, including four complete and one nearly complete circular genomes. All genomes exhibited canonical architectures, encoding Rep and Cap proteins and containing conserved HUH endonuclease and superfamily 3 helicase motifs. Phylogenetic analysis placed these viruses within the orders Rohanvirales, Ringavirales, Cirlivirales, and Cremevirales, representing multiple distinct evolutionary lineages. Genome-wide pairwise identity analysis showed that all identified viruses fell below established species- and genus-level thresholds, indicating that they represent novel taxa. Comparative analyses further revealed substantial divergence from known environmental and vertebrate-associated viruses.

**Discussion:**

These findings expand the known diversity of CRESS-DNA viruses in the human vaginal virome and highlight their broad evolutionary diversity. The detected viruses likely represent diverse ecological origins rather than stable host-specific infections, and no clear association with vaginitis was observed. This study provides new insights into the evolutionary landscape of CRESS-DNA viruses in the human reproductive tract and underscores the need for further investigation into their biological roles and potential health implications.

## 1 Introduction

Circular replication-associated protein (Rep)-encoding single-stranded DNA (CRESS-DNA) viruses are small, circular viruses with genomes typically ranging from 1.7 to 6.0 kilobases (kb) (Varsani et al., 2025; Zhao et al., 2019; Kazlauskas et al., 2018). They encode

at least two major open reading frames (ORFs): one for the Rep protein, which contains conserved HUH endonuclease and SF3 helicase domains essential for rolling-circle replication; and another for the highly diverse capsid (Cap) protein, which may have been acquired independently from multiple sources, including RNA viruses (Kazlauskas et al., 2019; Smiley et al., 2023; Tarasova and Khayat, 2023). CRESS-DNA genomes also contain a conserved nonanucleotide motif embedded within a stem-loop structure, which serves as the origin of replication (ori) (Zhao et al., 2019; Dai et al., 2024; Tarasova and Khayat, 2021). These viruses are ubiquitous and have been identified in a wide range of eukaryotic hosts and environmental reservoirs, such as seawater, sewage, and lakes (Chow and Suttle, 2015; Krupovic et al., 2020; Dayaram et al., 2016; Blinkova et al., 2009; Ng et al., 2012). More recently, they have also been detected in human swab samples, including respiratory secretions, feces, cerebrospinal fluid, blood, and pericardial fluid, although the causal associations with disease remain unclear (Prades et al., 2021; Zhang et al., 2025; Li et al., 2010; Smits et al., 2013). All CRESS-DNA viruses are currently classified under the phylum *Cressdnaviricota*, which as of now comprises two classes, 13 orders, 24 families, and 269 genera, with numbers continuing to increase (Krupovic et al., 2020). Beyond their taxonomic diversity, CRESS-DNA viruses are considered important models for studying virus evolution due to their modular genomes, evidence of horizontal gene transfer, and potential roles in shaping host-virus interactions (Kazlauskas et al., 2019; Desingu and Nagarajan, 2022).

The human vagina harbors a complex microbial ecosystem consisting of bacteria, archaea, fungi, and viruses, shaped by host- and environment-related factors such as ethnicity, age, pregnancy, and health status (Wang et al., 2025; Honorato et al., 2024). In healthy women, *Lactobacillus* species dominate the bacterial community, producing lactic acid that maintains an acidic environment and suppresses harmful microbes (Chee et al., 2020). The vaginal virome is less well characterized, but anelloviruses and papillomaviruses are consistently the most prevalent eukaryotic viruses, with higher abundance linked to cervical lesions and Lactobacillus-depleted communities (Chee et al., 2020; Li et al., 2023; Ravel et al., 2011). While human papillomavirus (HPV) has been extensively studied due to its established role in cervical carcinogenesis, knowledge of other eukaryotic viruses in the vaginal niche remains limited. Recent metagenomic studies have expanded this understanding, identifying CRESS-DNA viral genomes from the families *Genomoviridae* and *Smacoviridae* in vaginal secretions (Ramos et al., 2024; da Costa et al., 2024). These findings suggest that, beyond papillomaviruses and anelloviruses, the vagina may also serve as a reservoir for diverse CRESS-DNA viruses. However, their prevalence, host associations, and potential implications for reproductive health are still poorly understood.

In this study, we identified five novel CRESS-DNA viral genomes from female vaginal samples and performed a systematic analysis of their evolutionary relationships and potential biological significance. Our findings expand the known diversity of the vaginal virome and provide new insights into the evolution of CRESS-DNA viruses in the human reproductive tract.

## 2 Materials and methods

### 2.1 Sample collection and preparation

To investigate the vaginal virome and its potential association with vaginitis, women attending the Affiliated Hospital 6 of Nantong University, a tertiary care hospital in Yancheng, Jiangsu Province, China, were enrolled in January 2024. Participants were recruited after applying predefined exclusion criteria, including current pregnancy, medication-induced immunosuppression, antibiotic exposure within 1 month prior to sampling, and a history of cervical treatment or surgery. Based on colposcopic examination and microscopic evaluation of cervical secretions, individuals were categorized into a vaginitis group and a healthy control group. A total of 137 participants diagnosed with vaginitis and 130 healthy controls were included in the study. To facilitate downstream virome analyses and ensure comparable sequencing depth across pooled libraries, individuals within each clinical category were further randomly assigned into 12 subgroups, each comprising 10–12 participants. Pooling was conducted exclusively within clinical groups, and no additional stratification variables were applied prior to subgroup assignment. All swab samples were obtained from the Department of Clinical Laboratory, anonymized prior to analysis, and an exemption from informed consent was requested. The study protocol was approved by the Ethics Committee of Affiliated Hospital 6 of Nantong University (Approval No. 2024-34). Vaginal swabs were collected during gynecological consultations under speculum examination. After insertion of the speculum, swabs were used to sample the anterior and posterior vaginal fornices as well as cervical secretions. Each swab was immediately placed into a sterile collection tube and stored at 4 °C. For viral metagenomic analysis, swab tips were immersed in 0.5 ml Dulbecco's phosphate-buffered saline (DPBS), vortexed vigorously for 5 min, and incubated at 4 °C for 30 min. Supernatants were recovered by centrifugation at 15,000 × g for 10 min and stored at −80 °C until further processing.

### 2.2 Viral metagenomic sample processing and analysis

Approximately 45 μl of supernatant from each vaginal swab sample within the same subgroup was pooled together. Subsequently, the supernatant was filtered through a 0.45-μm filter (Millipore, Darmstadt, Germany) to remove eukaryotic cells, cell debris, and other large particles. Filtrates were then digested by DNase and RNase at 37 °C for 60 min. Total nucleic acids were then extracted using QIAamp MinElute Virus Spin Kit (Qiagen) according to the manufacturer's protocol. Nucleic acid samples were dissolved in DEPC treated water and RNase inhibitors were added. The enriched viral nucleic acid preparations from the respective pools were individually subjected to reverse transcription reactions using reverse transcriptase (PureScript Enzyme, Vazyme) and 100 pmol of random hexamer primers, followed by a single round of DNA synthesis using Klenow fragment polymerase (New England BioLabs). A total of 24 sequencing libraries were constructed using the TruePrep DNA Library Prep Kit (Vazyme) and sequenced on an Illumina NovaSeq 6000 platform using paired-end 150 bp reads (PE150), with an average library insert size of approximately 300 bp. As a negative control, a sterile swab moistened with ddH_2_O was processed alongside the clinical samples under identical experimental conditions, including nucleic acid extraction, library preparation, and sequencing. Sequencing of the control library yielded only a negligible number of reads, indicating minimal background contamination during sample processing and sequencing. To minimize host contamination, we downloaded the human reference genome (*Homo sapiens*, GCF_000001405.40) from NCBI and used Bowtie2 v2.4.5 (Langmead and Salzberg, 2012) for alignment and removal of potential host sequences from the 24 libraries. Primers and low-quality reads were trimmed using fastp v1.0.1 (Chen, 2025) with default settings (Supplementary Table 1). Paired-end reads from each pooled library were assembled independently. Initial *de novo* assembly was performed using MEGAHIT v1.2.9 (Li et al., 2016) with default parameters. To reduce false negatives and recover fragmented or circular viral genomes, unmapped reads and contigs shorter than 500 bp were subjected to additional semi-automated *de novo* assembly using the *De novo* assembler implemented in Geneious Prime (https://www.geneious.com). After reassembly, contigs longer than 1,500 bp were retained for downstream analyses. Contigs containing apparent frame shifts or assembly artifacts were manually inspected and removed.

### 2.3 Characterization and annotation of viral genomes

Quality-controlled reads and assembled contigs were aligned against the non-redundant protein (nr) database using BLASTx implemented in DIAMOND v2.0.15 (Buchfink et al., 2021) with an *E*-value threshold of < 10^−5^, and sequences with best hits to viral proteins were classified as viral, followed by taxonomic assignment using TaxonKit (Shen and Ren, 2021). To evaluate genome completeness and validate circularity, contigs were imported into Geneious Prime for manual curation and reference-based read mapping using the Low Sensitivity/Fastest setting. Read mapping depth and coverage continuity were examined to confirm complete or near-complete genome structures. Circular genomes were identified based on evidence of terminal redundancy and consistent read coverage across contig boundaries. Potential chimeric contigs were excluded through a combination of manual inspection, detection of inconsistent read-mapping patterns, and sequence clustering. To exclude potential vector contamination, sequences were screened using VecScreen (https://www.ncbi.nlm.nih.gov/tools/vecscreen) and then clustered at 95% nucleotide identity and 90% coverage using MMseqs2 (–*k 0* –*e 0.001 –min-seq-id 0.95* –*c 0.9 –cluster-mode 0*) (Mirdita et al., 2019). Viral genomes were curated and characterized based on consistent criteria, including evidence of circular structure or terminal redundancy, the presence of at least two major ORFs encoding Rep and Cap proteins, conserved Rep domains (HUH endonuclease motifs and SF3 helicase motifs), genome length >1.5 kb, and genomic architecture consistent with representative CRESS-DNA taxa. Putative ORFs were predicted in Geneious Prime using a minimum size threshold of 100 bp with an ATG start codon, and annotations were assigned using the Conserved Domain Database (CDD v3.21, NCBI) (Wang et al., 2023).

### 2.4 Phylogenetic analysis

Representative Rep amino acid sequences from the identified CRESS-DNA viruses were aligned with reference sequences using Clustal Omega v1.2.2 (Sievers et al., 2011). The alignments were subsequently inspected and manually trimmed to remove ambiguous or poorly aligned regions. Context sequences for phylogenetic analyses were retrieved from GenBank according to the latest ICTV taxonomy (https://ictv.global/vmr), and the most similar RefSeq sequences identified by BLASTn but absent from ICTV references were also included. Maximum likelihood tree were inferred with IQ-TREE v2.1.4 (Minh et al., 2020), applying the best-fit model determined by ModelFinder (Kalyaanamoorthy et al., 2017). Branch support was assessed with 1,000 ultrafast bootstrap replicates (Hoang et al., 2018), and the resulting tree was visualized in iTOL (https://itol.embl.de/). The tree was rooted using sequences from the class *Repensiviricetes* as outgroups, which are phylogenetically distinct from the focal viral lineages.

### 2.5 Pairwise sequence identity analysis

Pairwise nucleotide identity comparisons among all identified CRESS-DNA viruses were calculated using the Sequence Demarcation Tool (SDT v1.3) with the MAFFT alignment option (Muhire et al., 2014). The datasets used for SDT analysis comprised the newly identified CRESS-DNA viral genomes from this study together with representative reference genomes retrieved from GenBank based on their phylogenetic placement. Each sequence set was aligned within SDT, and identity matrices were generated to assist in species-level classification and similarity assessment.

## 3 Results

### 3.1 Viral metagenomic overview

**Figure 1:**
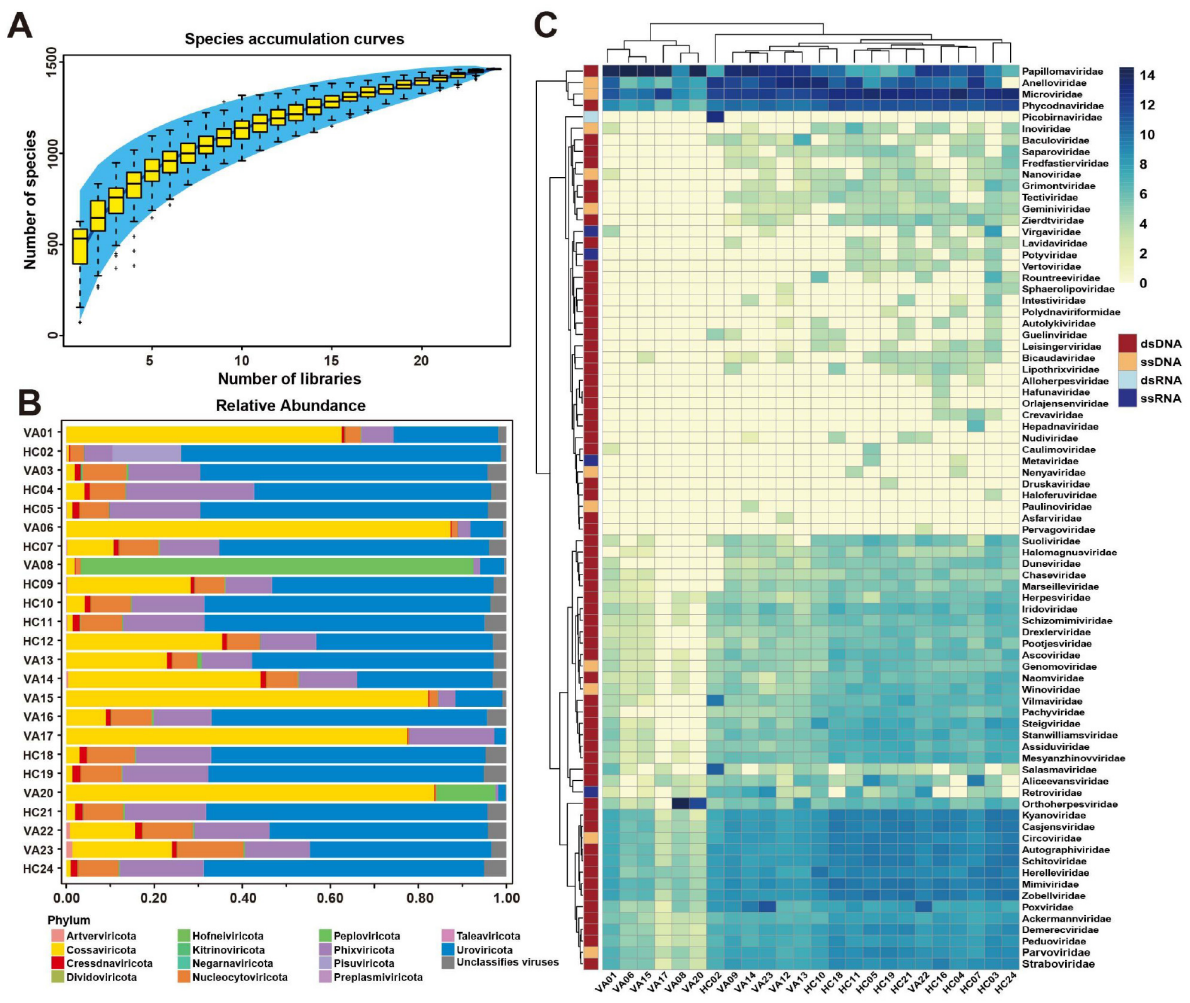
Viral metagenomic overview of the 24 libraries. Analyses were performed based on viral contigs >1,500 bp identified in this study. **(A)** Accumulation curve of viral contigs in this study. Error bars represent the range, and the blue area in the background represents the 95% confidence interval. **(B)** Bar graphs showing the relative proportion and taxonomy based on viral Phyla. **(C)** Heat map representing the viral contigs of each viral family of each library on a log10 scale. Viral genome types and viral families are annotated with corresponding colors (see color legend).

Metagenomic sequencing of the 24 vaginal swab libraries generated between 6.13 and 36.61 million paired-end reads per library (median: 19.24 million reads). After removal of host-derived sequences and quality control, 2.68–10.67 million reads per library were retained as high-quality non-host reads (median: 7.16 million reads). Based on DIAMOND BLASTx annotation against the nr database, viral reads ranged from 70,059 to 3,007,940 reads per library (median: 258,478 reads), accounting for 0.77%−30.48% of total reads (median: 3.57%; Supplementary Table 1). Species accumulation curves demonstrated that the sequencing depth was sufficient to capture the majority of viral diversity across the 24 vaginal libraries, with the number of unique positively identified species approaching a plateau at approximately 1,500 (Figure 1A). Taxonomic profiling at the phylum level revealed that *Cossaviricota* was the dominant lineage, accounting for 59.16% of all viral sequences, followed by *Uroviricota* at 16.66% (Figure 1B). Other phyla such as *Phixviricota* (9.88%), *Peploviricota* (9.98%), and *Nucleocytoviricota* (2.39%) were also detected at varying levels, reflecting substantial interindividual variation. Although *Cressdnaviricota* contributed only a small fraction of the total sequences (0.37% at the pooled dataset level), it was consistently detected in all libraries, with a mean relative abundance of 0.98% across individual libraries (IQR: 0.31%−1.47%; range: 0.05%−1.80%), underscoring its widespread presence in the vaginal virome (Supplementary Figure 1). Approximately 1% of contigs exhibited viral hallmarks but could not be assigned to any known phylum, suggesting the presence of highly divergent and as-yet unclassified viral lineages. At the family level, metagenomic sequencing identified viral reads spanning 79 families, including 63 families of double-stranded DNA (dsDNA) viruses, 11 single-stranded DNA (ssDNA) families, one double-stranded RNA (dsRNA) family, and four single-stranded RNA (ssRNA) families (Figure 1C). Notably, reads classified within *Papillomaviridae, Anelloviridae, Microviridae*, and *Phycodnaviridae* were significantly more abundant than those of other viral families. Several families of CRESS-DNA viruses, such as *Circoviridae, Genomoviridae*, and *Smacoviridae*, were also frequently detected, highlighting the widespread distribution of these small circular ssDNA viruses in the vaginal environment. Importantly, individual libraries displayed distinct viral community structures, with some dominated by a single phylum while others exhibited more balanced viral assemblages, reflecting heterogeneity in host-virus and virus-microbe interactions across individuals.

### 3.2 Identification of novel CRESS-DNA viruses in the human vagina

**Figure 2:**
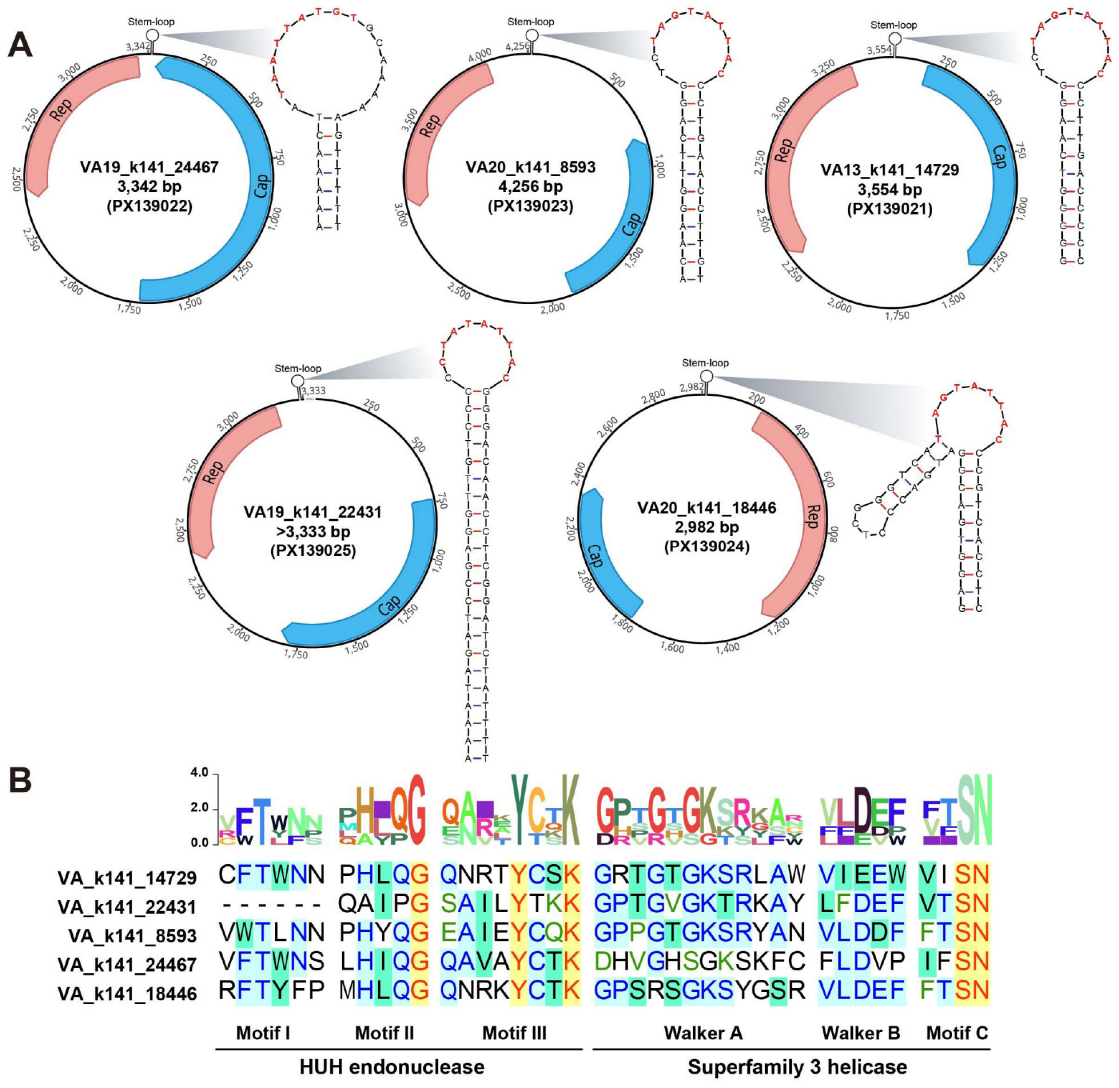
Genomic characterization and distribution of identified novel CRESS-DNA viruses. **(A)** Genome schematic organizations of the human-associated CRESS-DNA viruses sequenced in this study. **(B)** Identification of the HUH endonuclease domain and superfamily 3 helicase domain in the Rep protein.

A total of four complete and one nearly complete circular viral genomes were identified, and their genomic characteristics are summarized in Table 1. The genome sizes of these human vagina-associated CRESS-DNA viruses ranged from 2,982 to 4,256 bp, with G+C contents varying between 37.10% and 50.40%. These viruses exhibited the typical circular genomic organization characteristic of CRESS-DNA viruses, encoding two major genes: the replication-associated protein (*rep*) and the capsid protein (*cap*). Notably, two isolates (VA13_k141_14729 and VA19_k141_22431) displayed a classical ambisense genomic organization, in which the *rep* and *cap* genes are encoded on opposite strands of the double-stranded DNA replicative intermediate. In contrast, the remaining three genomes exhibited the typical monosense arrangement, with both genes oriented in the same direction (Figure 2A). The predicted Rep proteins ranged from 283 to 358 amino acids (aa), and Cap proteins from 193 to 559 aa. BLASTp analysis demonstrated that Rep sequences shared 44.91–97.40% identity with known CRESS-DNA viruses, while Cap proteins exhibited broader and generally lower identity levels (28.02–92.33%), consistent with the high structural variability typical of CRESS-DNA capsid proteins. All genomes harbored a predicted stem-loop structure within the intergenic region, though the structural characteristics differed among isolates (Figure 2A). A conserved nonanucleotide motif was located at the apex of the loop in all cases. Importantly, only VA20_k141_18446 exhibited distinct flanking inverted repeats, forming a more classical hairpin structure (Figure 2A). Domain analysis of Rep proteins revealed the complete sets of hallmark motifs of CRESS-DNA viruses (Zhao et al., 2019; Krupovic et al., 2020) (Figure 2B). The HUH endonuclease region contained conserved motif I, motif II, and motif III, while the superfamily 3 helicase (SF3H) region retained Walker A, Walker B, and Motif C, essential for NTP binding and helicase function. Despite minor lineage-specific sequence differences, these motifs were preserved across all identified genomes.

**Table 1:** Genomic features of genomes of CRESS-DNA viruses sequenced in this study.

Sequence ID	Accession	Size (nt)	GC (%)	Classification	Putative rep (aa)	Putative cap (aa)	Blastp hits on rep protein		Blastp hits on cap protein	
							Accession	Identity (%)	Accession	Identity (%)
VA13_k141_14729	PX139021	3,554	47.24	*Adamaviridae*	358	391	WPR18604.1	56.10	XOE96287.1	54.71
VA19_k141_22431	PX139025	>3,333	37.11	*Ringavirales* sp.	283	365	QMW68968.1	46.40	YP_009237572.1	41.72
VA19_k141_24467	PX139022	3,342	37.10	*Cremevirales* sp.	291	559	AUM61699.1	46.67	AXH74986.1	28.02
VA20_k141_8593	PX139023	4,256	42.13	*Cirlivirales* sp.	332	352	ADF80730.1	97.40	WOE49741.1	92.33
VA20_k141_18446	PX139024	2,982	50.40	*Cirlivirales* sp.	329	193	XII43102.1	44.91	UOF76666.1	42.78

### 3.3 Phylogenetic analysis

**Figure 3:**
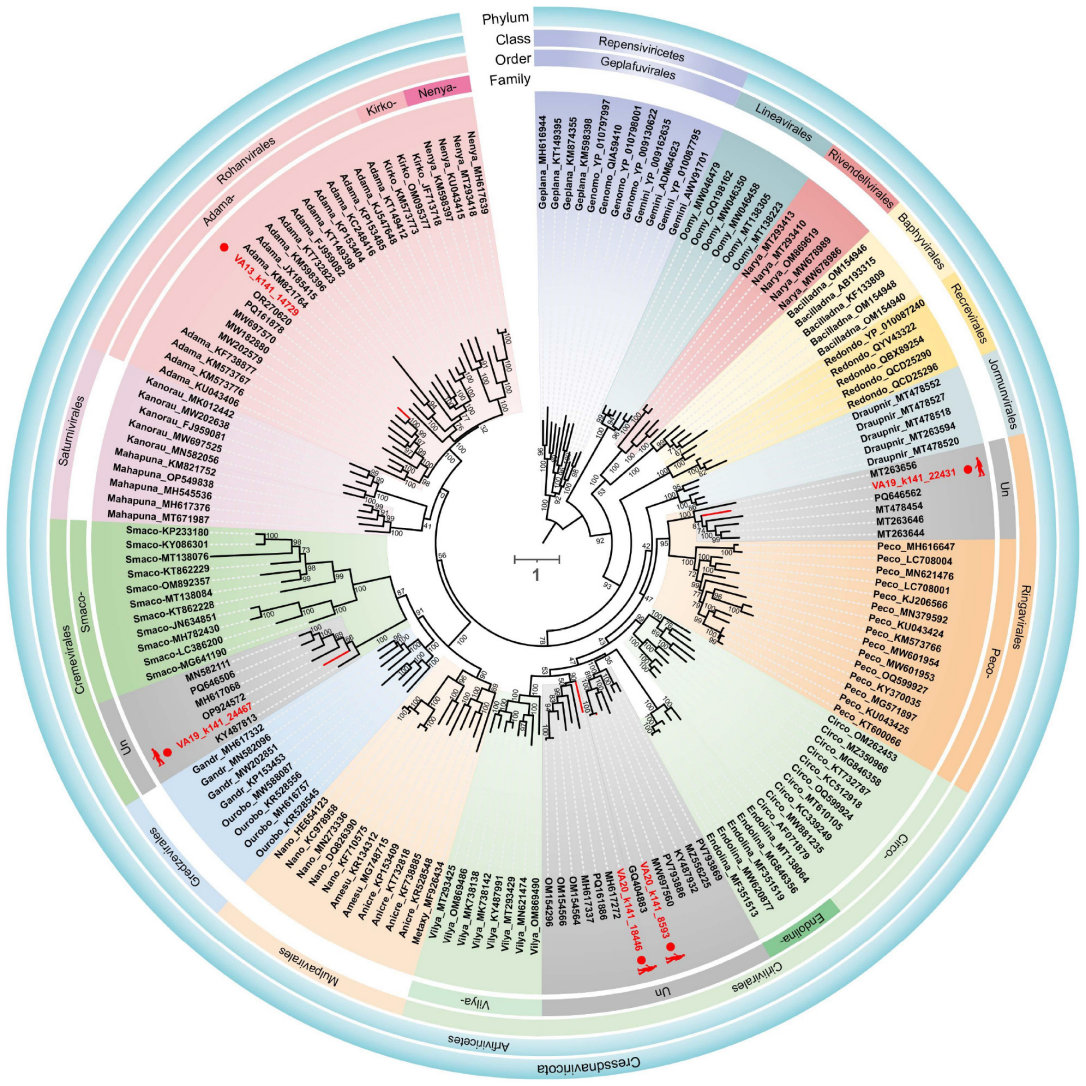
Maximum likelihood phylogenetic tree inferred from Rep proteins of members of the phylum *Cressdnaviricota*. The maximum likelihood phylogenetic tree was constructed using IQtree with automatic selection of the best-fit substitution model for a given alignment, which was Q.pfam + F + R6. Numbers at the nodes represent ultrafast bootstrap support values. The scale bar represents the number of substitutions per site. “Un” indicates unclassified families.

Based on the maximum likelihood phylogeny inferred from representative Rep protein sequences of the phylum *Cressdnaviricota*, the five newly identified viruses clustered into several distinct regions of the tree, corresponding to different evolutionary lineages within the phylum (Figure 3). Specifically, VA13_k141_14729 clustered within the family *Adamaviridae* and grouped with a sequence previously identified from the hemolymph of *Gonidea angulata*, sharing 56.10% amino acid sequence identity. In contrast, VA19_k141_24467 grouped with members of the order *Cremevirales* and formed a distinct lineage within this order, exhibiting a maximum amino acid sequence identity of 46.67% to viruses detected in wastewater. Likewise, VA19_k141_22431 clustered within the *Ringavirales* clade and grouped with its closest relative identified from aquatic environments. Furthermore, VA20_k141_8593 and VA20_k141_18446 clustered within the *Cirlivirales* clade, yet represented two separate and evolutionarily distinct lineages. Notably, four of the newly identified viruses could not be confidently assigned to any currently recognized viral families based on Rep-based phylogenetic placement alone. These phylogenetic placements were supported by high bootstrap values, indicating that the newly identified viruses represent phylogenetically diverse lineages within *Cressdnaviricota*.

**Figure 4:**
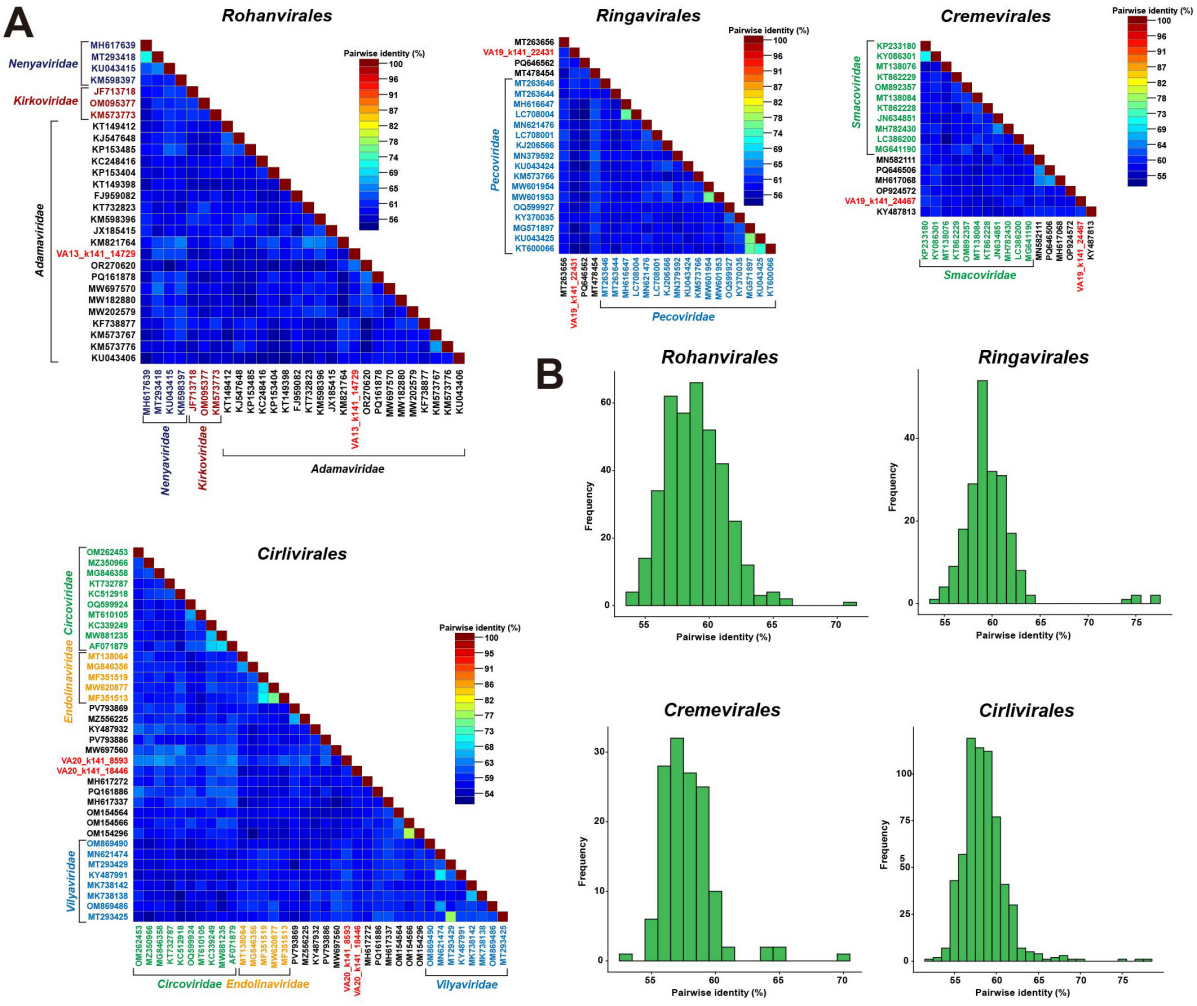
Pairwise sequence identity matrices of viruses belonging to the orders *Rohanvirales, Cremevirales, Ringavirales*, and *Cirlivirales*. **(A)** Pairwise identities were calculated using SDT v1.3 and are displayed as color-coded matrices, with the color scale indicating percentage sequence identity. Each matrix represents comparisons between the newly identified virus and representative reference genomes within the same order, encompassing one or more recognized families where applicable. **(B)** Histograms showing genome-wide pairwise nucleotide sequence identities between each newly identified virus and reference genomes within the corresponding order.

Pairwise sequence identity analysis based on whole-genome alignments showed that all five newly identified viruses shared genome-wide nucleotide identities below the species demarcation thresholds (approximately 77%−80%, depending on family) defined by ICTV classification criteria (Breitbart et al., 2017; Varsani and Krupovic, 2018; Krupovic and Varsani, 2022; Varsani and Krupovic, 2017). In combination with Rep-based phylogenetic analyses and genome organization features, genome-wide pairwise identity analyses further supported taxonomic placement at different ranks. Specifically, VA13_k141_14729 showed its highest nucleotide identity to members of the family *Adamaviridae*, yet remained below the established species demarcation threshold, supporting its classification as a novel species within this family (Figure 4A). In contrast, the remaining four viruses displayed uniformly low genome-wide nucleotide identities to all available reference genomes within their respective orders, forming distinct identity distributions shown by pairwise identity histograms (Figure 4B) that fall well below recognized species- and genus-level thresholds. Together with their Rep-based phylogenetic positions, these results indicate that these viruses represent highly divergent lineages within *Cressdnaviricota* that cannot be confidently assigned to any currently recognized species, genera, or families.

## 4 Discussion

In this study, we identified and characterized five novel CRESS-DNA viruses from human vaginal swab samples, thereby expanding the known diversity of this viral group. Phylogenetic analyses classified these viruses into the orders *Rohanvirales, Ringavirales, Cirlivirales*, and *Cremevirales*, indicating that evolutionarily distinct CRESS-DNA lineages can be present in the human vaginal environment. Consistent with their phylogenetic placement, these genomes exhibited canonical features of CRESS-DNA viruses, including conserved Rep protein motifs. Notably, CRESS-DNA viruses were detected in both the vaginitis group and healthy controls, and no specific viral lineage was exclusively associated with a particular clinical condition in this cohort. At present, we did not identify a disease-specific genomic signature or abundance pattern that would indicate a direct association between any individual CRESS-DNA virus and vaginitis. Similarly, although CRESS-DNA viruses have been detected in respiratory and oro-pharyngeal samples, including samples from individuals diagnosed with respiratory disorders or periodontal disease (Abbas et al., 2019; Taylor et al., 2022), no causal relationship has been established. Members of the phylum *Cressdnaviricota* include established animal pathogens, such as circoviruses infecting pigs and birds (Werling et al., 2021; Guo et al., 2022). However, clear evidence of human pathogenicity remains limited. In addition, several CRESS-DNA families, including *Genomoviridae* and *Smacoviridae*, are believed to infect fungi or archaea, suggesting that their detection in human samples may reflect associations with components of the microbial community rather than direct infection of human cells. These observations collectively suggest that CRESS-DNA viruses may exhibit a broader tissue and ecological distribution within the human body, and that their origins and transmission routes are likely more complex than currently understood.

Among the five newly identified CRESS-DNA viruses described here, four exhibited less than 60% amino acid sequence identity in the Rep protein compared with their closest known relatives, underscoring their substantial evolutionary divergence. One notable exception was VA20_k141_8593, which shared 97.40% Rep amino acid sequence identity with a virus previously identified from chimpanzee stool samples (Table 1). This high level of sequence identity may suggest a relatively recent common ancestry or a broader host-associated distribution for this lineage, although its exact host range and transmission dynamics remain unclear. Notably, the remaining four newly identified CRESS-DNA viruses clustered phylogenetically with viruses previously detected in environmental sources, including wastewater, as well as in samples associated with invertebrates. This pattern suggests that CRESS-DNA viruses could occupy a broad ecological range, with their detection in vaginal samples potentially reflecting various sources, rather than stable host-associated infection. One potential explanation is environmental exposure, although alternative origins, including translocation from other body sites, cannot be excluded. In this context, the likelihood that these viruses play a direct pathogenic role in humans appears limited. It should be noted that a spike-in positive control was not included in the metagenomic workflow of this study. Although a negative control was processed in parallel and showed negligible background contamination, the lack of a positive control limits our ability to directly evaluate the overall sensitivity of the sequencing and bioinformatic pipeline. Nevertheless, the relatively low sequence similarity of their capsid proteins to those of known viral species implies that these viruses may possess distinct structural features or host interaction strategies. Future studies should prioritize experimental assessment of their replication potential in human or microbial cell culture systems, together with large-scale population-based investigations and serological screening, to better characterize their distribution and biological relevance. In addition, systematic screening of diverse environmental metagenomic datasets, including aquatic systems and wastewater, will be important to determine whether these viruses are specifically associated with the vaginal niche or represent more broadly distributed environmental lineages.

Given the widespread distribution and high diversity of CRESS-DNA viruses, further research is needed to explore their potential role in shaping microbial communities and influencing human health. Understanding the interactions between these viruses and the host immune system, their co-infection patterns with other pathogenic viruses, and their transmission dynamics is crucial for elucidating their biological significance. Our study further reveals the previously unrecognized viral diversity within the human vaginal microbiome, highlighting the importance of continued metagenomic surveillance to more comprehensively characterize the human virome and assess its potential public health implications.

## Data Availability

The datasets presented in this study can be found in online repositories. The names of the repository/repositories and accession number(s) can be found below: https://www.ncbi.nlm.nih.gov/, PRJNA1170175.
