# Supplementary material for: Identification of novel CRESS-DNA viruses in the human vaginal microbiome
Source: Front Microbiol. 2026 Apr 17;17:1790643. doi: 10.3389/fmicb.2026.1790643 (PMC13133057; doi:10.3389/fmicb.2026.1790643)

Supplementary Figure 1. Relative abundance of viral phyla across metagenomic libraries.
Boxplots show the distribution of relative abundance (%) for viral phyla across 24 vaginal swab libraries, with the central line indicating the median, boxes representing the interquartile range, and whiskers denoting 1.5 × IQR. Non-zero values are shown as individual points.

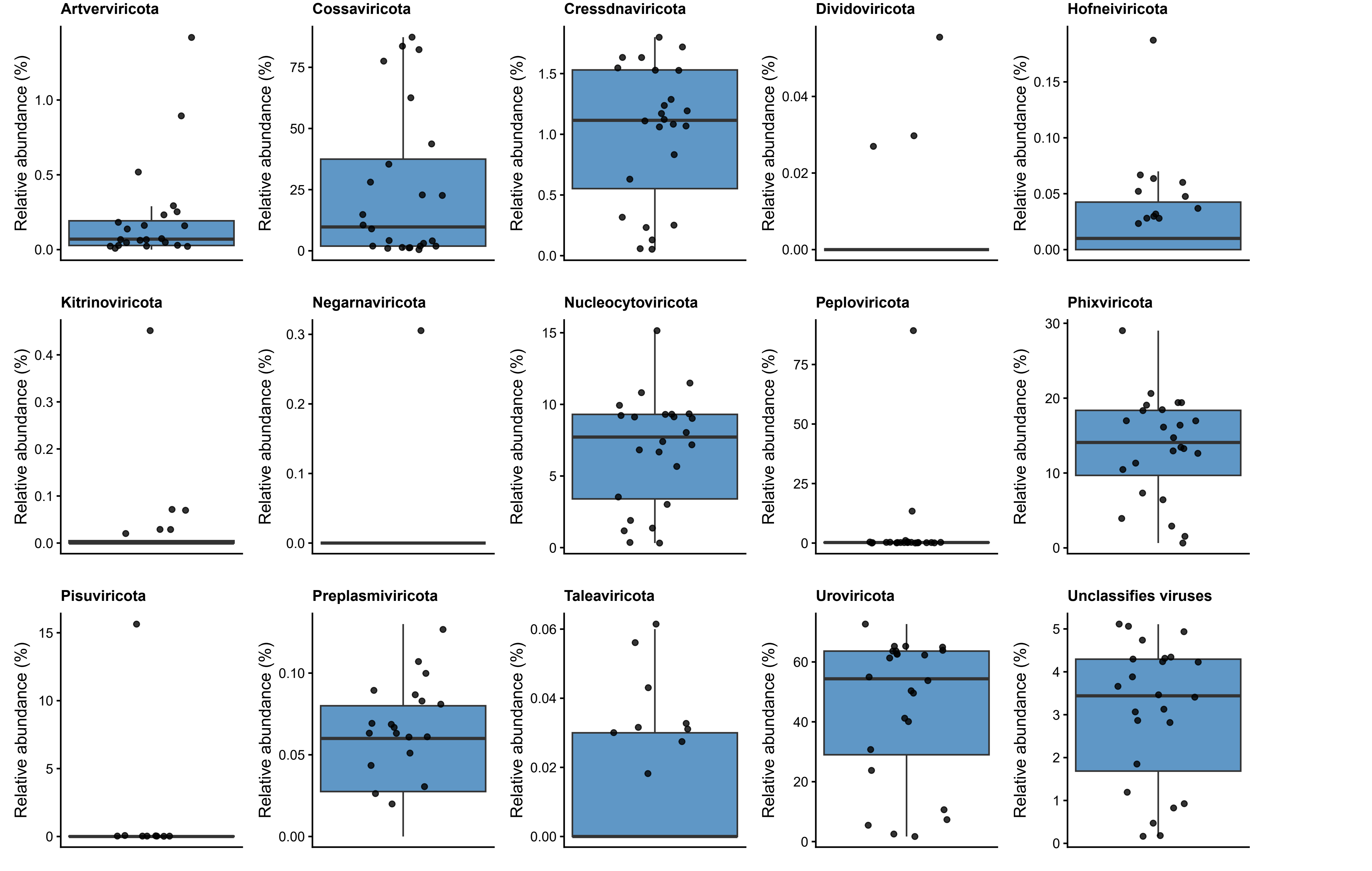

Supplement: Supplementary file 1 [file Figure_1.pdf]
